# Factors associated with neutralizing antibody levels induced by two inactivated COVID-19 vaccines for 12 months after primary series vaccination

**DOI:** 10.3389/fimmu.2022.967051

**Published:** 2022-09-09

**Authors:** Fuzhen Wang, Baoying Huang, Huakun Lv, Lizhong Feng, Weihong Ren, Xiaoqi Wang, Lin Tang, Qianqian Liu, Dan Wu, Hui Zheng, Zhijie An, Yao Deng, Li Zhao, Fei Ye, Wenling Wang, Hangjie Zhang, Shaoying Chang, Yuting Liao, Fengyang Chen, Lance E. Rodewald, George F. Gao, Zundong Yin, Wenjie Tan

**Affiliations:** ^1^ National Immunization Program, Chinese Center for Disease Control and Prevention, Beijing, China; ^2^ National Health Commission (NHC) Key Laboratory of Biosafety, Institute for Viral Disease Control and Prevention, Chinese Center for Disease Control and Prevention, Beijing, China; ^3^ Immunization Program Institute, Zhejiang Provincial Center for Disease Control and Prevention, Hangzhou, China; ^4^ Immunization Program Institute, Shanxi Provincial Center for Disease Control and Prevention, Taiyuan, China; ^5^ Xingtai Center for Disease Control and Prevention, Xingtai, China; ^6^ School of Public Health, Xiamen University, Xiamen, China; ^7^ Chinese Center for Disease Control and Prevention, Beijing, China

**Keywords:** SARS-CoV-2, COVID-19 vaccine, immunogenicity, immune persistence, influencing factors

## Abstract

**Background:**

BBIBP-CorV and CoronaVac inactivated COVID-19 vaccines are widely-used, World Health Organization-emergency-listed vaccines. Understanding antibody level changes over time after vaccination is important for booster dose policies. We evaluated neutralizing antibody (nAb) titers and associated factors for the first 12 months after primary-series vaccination with BBIBP-CorV and CoronaVac.

**Methods:**

Our study consisted of a set of cross-sectional sero-surveys in Zhejiang and Shanxi provinces, China. In 2021, we enrolled 1,527 consenting 18-59-year-olds who received two doses of BBIBP-CorV or CoronaVac 1, 3, 6, 9, or 12 months earlier and obtained blood samples and demographic and medical data. We obtained 6-month convalescent sera from 62 individuals in Hebei province. Serum nAb titers were measured by standard micro-neutralization cytopathic effect assay in Vero cells with ancestral SARS-CoV-2 strain HB01. We used the first WHO International Standard (IS) for anti-SARS-CoV-2 immunoglobulin (NIBSC code 20/136) to standardized geometric mean concentrations (IU/mL) derived from the nAb geometric mean titers (GMT over 1:4 was considered seropositive). We analyzed nAb titer trends using Chi-square and factors related to nAb titers with logistic regression and linear models.

**Results:**

Numbers of subjects in each of the five month-groupings ranged from 100 to 200 for each vaccine and met group-specific target sample sizes. Seropositivity rates from BBIBP-CorV were 98.0% at 1 month and 53.5% at 12 months, and GMTs were 25.0 and 4.0. Respective seropositivity rates from CoronaVac were 90.0% and 62.5%, and GMTs were 20.2 and 4.1. One-, three-, six-, nine-, and twelve-month GMCs were 217.2, 84.1, 85.7, 44.6, and 10.9 IU/mL in BBIBP-CorV recipients and 195.7, 94.6, 51.7, 27.6, and 13.4 IU/mL in CoronaVac recipients. Six-month convalescent seropositivity was 95.2%; GMC was 108.9 IU/mL. Seropositivity and GMCs were associated with age, sex, and time since vaccination.

**Conclusions:**

Neutralizing Ab levels against ancestral SARS-CoV-2 from BBIBP-CorV or CoronaVac vaccination were similar and decreased with increasing time since vaccination; over half of 12-month post-vaccination subjects were seropositive. Seropositivity and GMCs from BBIBP-CorV and CoronaVac six and nine months after vaccination were similar to or slightly lower than in six-month convalescent sera. These real-world data suggest necessity of six-month booster doses.

## Introduction

Vaccination is indispensable for reducing suffering and death from the COVID-19 pandemic. To date, there are five COVID-19 vaccines that have received conditional approval for market authorization in China by the National Medical Products Administration (NMPA). The vast majority of COVID-19 vaccine doses administered are Vero cell grown, whole virus, alum adjuvanted inactivated vaccine produced by Beijing Institute of Biological Products CO., LTD, SINOPHARM (BBIBP-CorV vaccine) and Sinovac Research & Development Co., Ltd (CoronaVac vaccine) (1, 2). To date, the World Health Organization (WHO) has listed eleven vaccines based on four technical platforms for Emergency Use (EUL), including inactivated virus vaccines, mRNA based vaccines, protein subunit vaccines, and viral vectored vaccines. WHO listed the two China-produced inactivated vaccines for emergency use in May (BBIBP-CorV) and June (CoronaVac) of 2021 (3, 4). By October 2021, BBIBP-CorV and CoronaVac accounted for nearly half of COVID-19 vaccines doses administered globally (5).

Phase 1, 2, and 3 clinical trials demonstrated acceptable immunogenicity and safety of both vaccines. Studies of antibody response kinetics and immune persistence showed that neutralizing antibody (nAb) levels from all COVID-19 vaccines wane, regardless of technological platform (6–9), and that waning is associated with decreased protection. Before and after large-scale use in populations, kinetics and persistence of nAb from inactivated vaccines showed immune attenuation (10, 11), but data are limited, especially in China. Some immune persistence studies are challenged by non-standardized laboratory methods and high background rates of natural infection (12–14).

There are no published population-based real-world studies that have explored antibody response kinetics and immune persistence more than 6 months after China’s large-scale vaccination campaign, despite the administration of over 2.8 billion doses of COVID-19 vaccines by the end of 2021 (15). No data have been reported on the influence of population factors on immune persistence. It is important to document immunity by time since vaccination with real world data in China to provide evidence supporting vaccination strategy refinement in this infection-naïve population where immunity against SARS-CoV-2 comes almost solely from vaccines.

We conducted a real-world, observational, cross-sectional study of healthy adults aged 18-59 years to assess immune marker persistence during the first year after full-series vaccination with BBIBP-CorV and CoronaVac COVID-19 vaccines in China using standardized laboratory testing methods. We report results of our study.

## Methods

### Setting, design, and participants

The study was set in two provinces in the south and north of mainland China (Zhejiang and Shanxi) and was initiated after nationwide containment of SARS-CoV-2 in April 2020 and completed during sustained implementation Dynamic COVID-Zero policy that prevents local transmission. COVID-19 vaccination started in the summer of 2020 during a period of emergency use authorization, and a nationwide campaign started in December 2020. During the study period, March 2021 to December 2021, there was almost no transmission of SARS-CoV-2 in China other than small, rapidly contained import-related outbreaks, none of which were in the study areas. Lack of local transmission allowed observation of immune persistence following vaccination in the absence of exogenous boosting by SARS-CoV-2 exposure.

The design was a set of cross-sectional sero-surveys of people who had been vaccinated in pre-specified times before study enrollment. Participants were individuals aged 18-59 years who were fully vaccinated with two doses of an inactivated vaccine, with inter-dose intervals of 3 to 4 weeks and the second dose administered 1, 3, 6, 9, or 12 months prior to enrollment and obtaining blood samples. This was not a longitudinal survey, as the subjects were different at each evaluation month. Exclusion criteria were history of infection with SARS-CoV-2, improper inter-dose interval, receipt of vaccine other than BBIBP-CorV or CoronaVac, history of using blood products or immunosuppressive drugs, and history of severe adverse reaction following immunization.

The study vaccines were BBIBP-CorV and CoronaVac; both are ancestral strain, whole-virus, β-propiolactone-inactivated, aluminum hydroxide adjuvanted, liquid COVID-19 vaccines. These two inactivated vaccines have been conditionally approved for individuals 18 years and older by the National Medical Products Administration (NMPA), the vaccine regulatory authority of China, prior to the start of the study. The recommended immunization schedule for both vaccines is two doses *via* intramuscular injection with an interval of 3-8 weeks.

In addition to sera from the vaccinated subjects, we obtained convalescent sera following a COVID-19 outbreak in Hebei province. On January 3, 2021, Nangong City, part of Xingtai City, Hebei Province, reported its first symptomatic case of COVID-19, leading to an outbreak in Xingtai, representing local transmission caused by an imported case (11). All cases were infected the SARS-CoV-2 D614G variant; 10.5% of infections were asymptomatic and the 89.5% of cases that were symptomatic were of mild or moderate severity. Convalescent sera were obtained from cases in this outbreak for comparison with sera from vaccinated subjects in our survey.

### Enrollment and sample collection

Provincial centers of diseases control and prevention (CDCs) recruited subjects using identification and contact data from their immunization information systems. CDCs listed potentially-eligible subjects whose vaccinations were managed in the jurisdiction of the CDC. Trained staff called potentially-eligible subjects and explained the study purpose, protocol, implementation process, information and specimen collection procedures, and risks and benefits over the phone. Individuals willing and eligible to participate became study subjects, representing convenience samples of individuals who had been vaccinated at just short of one of the five pre-determined intervals prior to study participation.

We made separate estimates of sample sizes for each of the two vaccines in two age groups (18-39 years and 40-59 years), and for five discrete times since administration of the second dose (1, 3, 6, 9, and 12 months) – a total of 20 groups. We based sample sizes using α=0.05, β=0.20, two-sided tests, with expert-opinion estimates of expected seropositive by time since vaccination of 98% at 1 month, 90% at 3 months, 80% at 6 months, 70% at 9 months, and 60% at 12 months and effect sizes of no more than 10 percentage point differences from expected values. The required sample sizes for each vaccine were 95, 108, 153, 182, and 194 subjects, respectively; these required sample sizes were adjusted upward to target sample sizes of 100, 110, 160, 190, and 200 subjects for each vaccine – a total target sample size of 1,520 subjects.

Participants had been vaccinated over a range of times prior to enrollment. Blood draws were scheduled for the next discrete time since vaccination: 1, 3, 6, 9 or 12 months after the administration of a subject’s second dose. We administered a questionnaire survey at blood drawing visits to gather demographic information.

**Figure 1:**
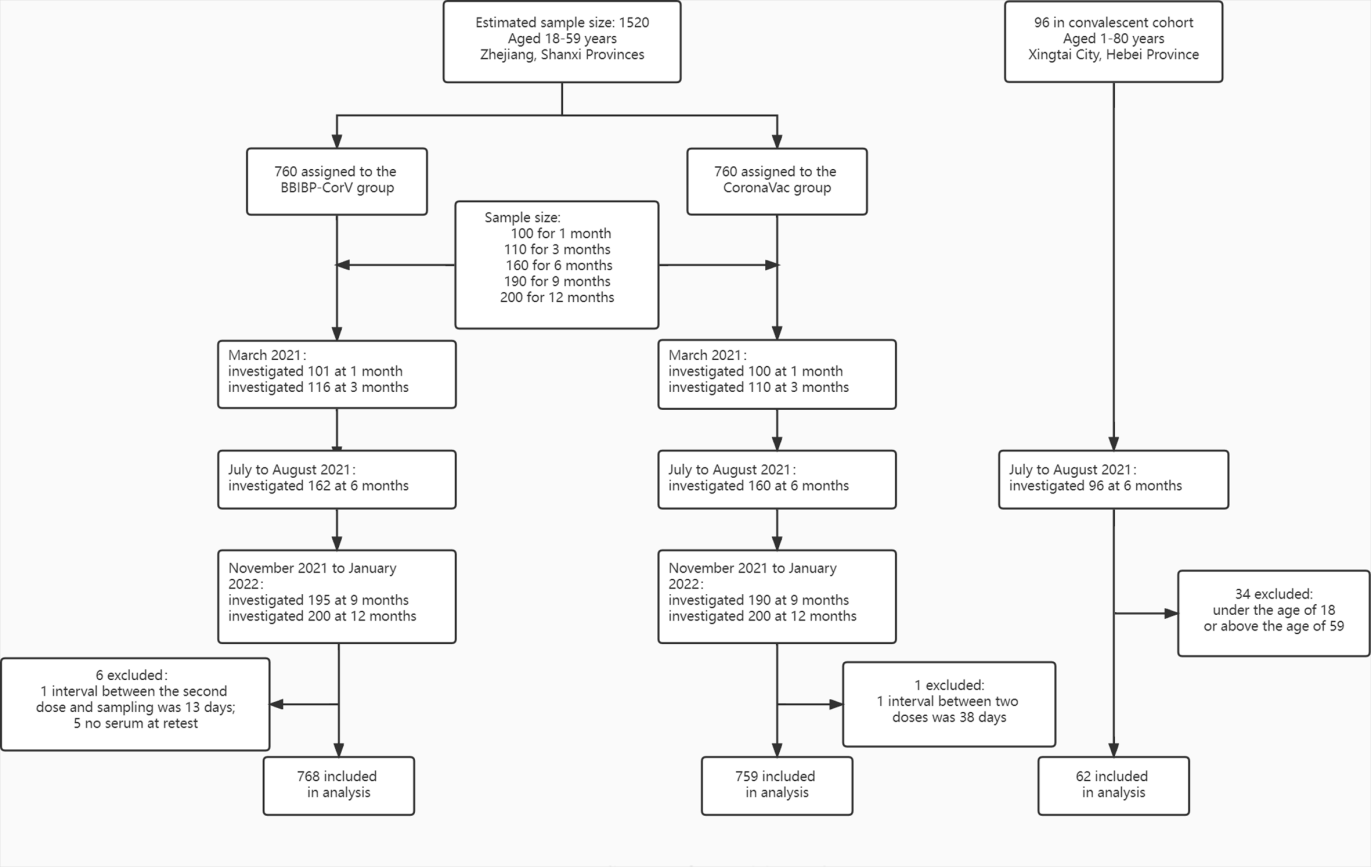
Study flow diagrams for participants by specific vaccine.

We obtained six-month convalescent sera and demographic information from individuals, 18-59-years-of-age, who had been infected in a COVID-19 outbreak in Xingtai city of Hebei province (**Figure 1**).

### Laboratory testing and standardization

Neutralization assays were conducted in a BSL-3 laboratory. Serum nAb responses were assessed by reduction of cytopathic effect (CPE) in Vero cells with infectious ancestral SARS-CoV-2 strain 19nCoV-CDC-Tan-HB01 (HB01). Briefly, serum was inactivated at 56°C for 30 minutes and successively diluted from 1:4 to the required concentration in 2-fold series. An equal volume of challenge virus solution containing 100 CCID_50_ virus was added. After neutralization in a 37°C incubator for 2 hours, a 1.5-2.5×10^5^/mL cell suspension was added to the wells; cytopathic effect was assessed 4 days after infection. Neutralization titers (NT_50_) were expressed as the reciprocal of the highest dilution protecting 50% of the cells from the virus challenge.

To facilitate comparison of SARS-CoV-2 neutralization assay data from multiple assay formats and vaccines, we used WHO international standard (IS) and an internal neutralization standard. The WHO 1^st^ IS for antiserum to SARS-CoV-2 (NIBSC code 20/136) was obtained from National Institute for Biological Standards and Control (NIBSC). The internal neutralization standard ‘R1’ was generated in-house by Beijing Minhai Biotechnology Co., Ltd. by pooling a selection of SARS-CoV-2 RBD protein immunized goat sera. All neutralization standards were run in triplicate on the SARS-CoV-2 neutralizing assay described above. The internal reference was calibrated according to the WHO 1^st^ IS for anti-SARS-CoV-2 immunoglobulin; test samples compared to the IS can be expressed in IU/mL by calculation: GMT of test samples/(GMT of SARS-CoV-2 IS/1000) = IU/mL. The calibrated potency of internal standard ‘R1’ was 16,734 IU/mL. The internal reference was included in every experiment and used for correction of tested sample results. To convert sample neutralization titers into international units, the neutralization dilution values of the sample were divided by the neutralization of the internal standard run during the same experiment and then multiplied by the calibrated potency of the respective internal standards in international units. Use of the reference standard sera allowed neutralization assay outputs to be converted to international units per milliliter (IU/mL).

### Statistical analyses

We used mean ± standard deviation (SD) for age, medians, and interquartile ranges (IQR) for other continuous variables, and numbers (percentages) for categorical variables. Outcome variables were immune markers and seropositivity (GMT over 1:4). Immunogenicity was expressed by nAb seroconversion percentage, geometric mean titers (GMT), and geometric mean concentrations (GMC, IU/mL) referenced to WHO, with associated 95% confidence intervals (CI). Antibody titers were log-transformed to calculate GMT per group. We assessed trends over time with the Cochran-Armitage trend test, and used the Kruskal Wallis test to compare GMCs of convalescent sera with GMCs of sera from the vaccinated subjects. To determine factors related to nAb titers, we used logistic regression analysis and a linear model with gender, age group, inter-dose interval, and manufacturer as covariates. All analyses were done using R (version 4.1.0). Statistical tests were two-sided, and we considered *P* values of 0.05 or less as statistically significant.

## Results

### Participants

We recruited 1,527 individuals who met all inclusion criteria and no exclusion criteria and who had received two doses of inactivated COVID-19 vaccine in October 2020 or later. Among these individuals, 768 had been vaccinated with BBIBP-CorV and 759 had been vaccinated with CoronaVac. Participants ranged in age from 18 to 59 years, with a mean age of 39.3 (sd 10.7 years); 52.5% of participants were female; 9.0% had a BMI ≥28.0 kg/m^2^; 8.9% had ≥1 underlying comorbidities (most commonly hypertension and diabetes); 480 (31.4%) were vaccinated with an inter-dose interval of 21-27 days and 1,047 (68.6%) with an inter-dose interval of 28-35 days; no participants had been diagnosed with COVID-19 before the study (**Table 1**).

**Table 1:** Participant characteristics and nAb responses to inactivated COVID-19 vaccine.

Variable	1 month after 2^nd^ dose				3 months after 2^nd^ dose				6 months after 2^nd^ dose				9 months after 2^nd^ dose				12 months after 2^nd^ dose			
	N	(+)%*	GMT (95%CI)	GMC (95%CI)	N	(+)%	GMT (95%CI)	GMC (95%CI)	N	(+)%	GMT (95%CI)	GMC (95%CI)	N	(+)%	GMT (95%CI)	GMC (95%CI)	N	(+)%	GMT (95%CI)	GMC (95%CI)
**BBIBP-CorV(N=768)**																				
Total	100	98.0	25.0 (21.3-29.4)	217.2 (178.5-264.3)	111	95.5	10.2 (8.7-12.0)	84.1 (68.1-103.9)	162	90.7	11.6 (10.1-13.3)	85.7 (69.1-106.3)	195	85.6	7.9 (6.9-9.0)	44.6 (36.1-55.0)	200	53.5	4.0 (3.5-4.4)	10.9 (8.7-13.8)
Sex																				
Male	51	100.0	25.3 (20.4-31.4)	229.7 (185.1-285.1)	52	90.4	7.5 (5.9-9.5)	55.4 (38.4-79.8)	80	90.0	11.2 (9.2-13.6)	81.6 (59.6-111.9)	101	84.2	6.1 (5.3-7.1)	32.9 (25.2-42.9)	99	44.4	3.3 (2.9-3.7)	7.6 (5.6-10.3)
Female	49	95.9	24.8 (19.4-31.8)	204.9 (145.9-287.7)	59	100.0	13.4 (11.0-16.3)	121.7 (99.9-148.1)	82	91.5	11.9 (9.8-14.5)	89.8 (66.4-121.4)	94	87.2	10.4 (8.4-12.8)	61.8 (44.9-85.0)	101	62.4	4.8 (4.0-5.7)	15.6 (11.2-21.8)
Age group (at vaccination, years)																				
18-29	21	100.0	36.2 (29.0-45.4)	329.5 (263.3-412.3)	26	92.3	11.1 (7.6-16.4)	85.5 (49.1-148.8)	43	95.3	14.4 (10.9-19.0)	118.1 (81.7-170.7)	53	88.7	8.7 (6.6-11.5)	47.1 (31.8-69.9)	56	62.5	4.7 (3.8-5.9)	15.5 (9.9-24.3)
30-39	29	100.0	22.6 (17.9-28.5)	205.4 (162.8-259.1)	28	96.4	9.8 (7.5-12.9)	82.5 (56.8-119.8)	40	90.0	9.9 (7.7-12.6)	71.8 (46.9-109.9)	47	91.5	10.0 (7.9-12.6)	67.7 (47.0-97.5)	44	63.6	4.8 (3.7-6.2)	16.0 (9.6-26.5)
40-49	25	100.0	25.6 (17.5-37.5)	233.2 (159.3-341.2)	34	100.0	10.2 (7.6-13.7)	92.8 (68.9-125.0)	39	89.7	10.0 (7.6-13.0)	72.1 (46.3-112.4)	49	81.6	6.5 (5.0-8.4)	34.3 (22.1-53.4)	53	49.1	3.5 (2.9-4.2)	8.8 (5.7-13.5)
50-59	25	92.0	20.2 (13.3-30.8)	152.1 (81.8-282.8)	23	91.3	9.8 (6.6-14.5)	73.2 (40.6-132.3)	40	87.5	12.4 (8.9-17.3)	85.5 (50.8-144.1)	46	80.4	6.8 (5.1-9.2)	36.0 (22.0-58.8)	47	38.3	3.1 (2.5-3.9)	6.5 (4.1-10.2)
Obesity (BMI≥28 kg/m^2^)																				
Yes	13	100.0	22.1 (13.5-36.3)	201.3 (122.8-330.0)	11	90.9	10.0 (6.0-16.7)	74.2 (30.6-179.8)	12	91.7	9.8 (5.7-16.7)	74.1 (32.0-171.8)	22	86.4	7.7 (5.2-11.6)	40.9 (21.9-76.7)	23	34.8	2.9 (2.2-3.7)	5.6 (3.0-10.6)
No	87	97.7	25.5 (21.5-30.3)	219.7 (176.9-272.8)	100	96.0	10.2 (8.6-12.2)	85.3 (68.5-106.2)	150	90.7	11.7 (10.1-13.5)	86.7 (69.2-108.6)	173	85.5	7.9 (6.9-9.1)	45.1 (36.0-56.4)	177	55.9	4.1 (3.7-4.6)	11.9 (9.3-15.2)
Chronic conditions																				
Yes	5	100.0	26.3 (11.6-59.5)	239.1 (105.7-541.1)	11	90.9	10.9 (4.7-25.3)	81.2 (26.6-247.9)	18	94.4	12.3 (8.5-17.9)	99.3 (56.2-175.4)	17	76.5	8.2 (4.2-16.0)	36.9 (13.3-102.1)	19	42.1	3.6 (2.3-5.5)	8.0 (3.4-18.7)
No	95	97.9	25.0 (21.1-29.5)	216.1 (176.1-265.3)	100	96.0	10.1 (8.7-11.9)	84.5 (68.5-104.2)	144	90.3	11.5 (9.9-13.3)	84.1 (66.6-106.3)	178	86.5	7.9 (6.9-9.0)	45.4 (36.7-56.1)	181	54.7	4.0 (3.6-4.5)	11.3 (8.9-14.4)
Interval between 2 doses (days)																				
21-27	96	97.9	24.5 (20.8-28.9)	211.9 (173.1-259.3)	96	94.8	10.2 (8.6-12.2)	83.0 (65.4-105.2)	23	100.0	13.6 (9.5-19.5)	123.6 (86.3-177.1)	22	90.9	7.9 (5.3-11.7)	50.4 (28.3-90.0)	34	58.8	4.2 (3.2-5.4)	12.7 (7.2-22.4)
28-35	4	100.0	43.4 (16.7-112.9)	394.3 (151.5-1026.3)	15	100.0	10.1 (6.9-15.0)	92.0 (62.3-136.0)	139	89.2	11.3 (9.7-13.1)	80.6 (63.2-102.9)	173	85.0	7.9 (6.8-9.1)	43.9 (35.0-55.0)	166	52.4	3.9 (3.5-4.4)	10.6 (8.2-13.7)
**CoronaVac(N=759)**																				
Total	100	90.0	20.2 (16.3-24.9)	195.7 (139.5-274.5)	110	98.2	14.7 (12.7-17.0)	94.6 (79.8-112.1)	159	85.5	7.8 (6.9-8.8)	51.7 (41.3-64.9)	190	80.5	6.0 (5.3-6.7)	27.6 (22.5-33.9)	200	62.5	4.1 (3.7-4.5)	13.4 (10.8-16.6)
Sex																				
Male	49	91.8	21.4 (15.8-29.0)	217.6 (136.9-345.7)	54	96.3	12.9 (10.5-15.9)	80.2 (61.3-105.1)	80	81.2	6.8 (5.7-8.1)	41.1 (29.1-58.0)	85	81.2	6.4 (5.3-7.7)	29.7 (21.7-40.7)	75	61.3	3.8 (3.3-4.4)	12.1 (8.6-17.0)
Female	51	88.2	19.0 (14.0-25.9)	176.8 (106.5-293.3)	56	100.0	16.6 (13.5-20.5)	110.9 (89.9-136.7)	79	89.9	9.0 (7.6-10.6)	65.4 (48.9-87.4)	105	80.0	5.7 (4.9-6.6)	26.0 (19.8-34.1)	125	63.2	4.3 (3.8-4.9)	14.2 (10.8-18.8)
Age group (at vaccination, years)																				
18-29	19	89.5	24.0 (13.8-41.6)	229.8 (95.2-554.4)	19	100.0	13.6 (9.7-19.1)	90.5 (64.4-127.4)	35	94.3	11.3 (8.8-14.5)	90.7 (61.8-133.2)	40	85.0	5.9 (4.7-7.3)	29.5 (19.8-44.1)	36	72.2	4.4 (3.5-5.5)	17.2 (10.6-27.9)
30-39	31	83.9	21.9 (13.6-35.4)	182.1 (82.8-400.3)	35	100.0	17.4 (12.9-23.4)	115.9 (85.9-156.3)	43	93.0	9.5 (7.8-11.7)	74.4 (52.7-104.9)	53	71.7	5.3 (4.3-6.6)	20.7 (13.4-31.9)	68	55.9	3.7 (3.1-4.4)	10.7 (7.3-15.6)
40-49	24	91.7	16.9 (10.7-26.7)	171.3 (86.6-338.7)	22	100.0	16.0 (12.4-20.8)	107.0 (82.6-138.6)	51	86.3	6.7 (5.5-8.2)	44.9 (30.6-65.8)	66	86.4	6.5 (5.3-7.9)	33.3 (24.1-46.1)	63	65.1	4.6 (3.8-5.5)	15.7 (10.5-23.4)
50-59	26	96.2	18.9 (14.6-24.6)	214.5 (139.9-328.9)	34	94.1	12.2 (9.2-16.2)	72.6 (48.9-107.7)	30	63.3	5.0 (3.7-6.8)	20.3 (10.2-40.4)	31	77.4	6.4 (4.6-8.9)	27.8 (15.5-49.7)	33	60.6	3.8 (3.0-4.9)	12.0 (7.0-20.7)
Obesity (BMI≥28 kg/m^2^)																				
Yes	9	66.7	13.7 (4.5-42.2)	73.9 (9.2-596.0)	5	100.0	13.2 (7.2-24.0)	87.7 (48.1-159.7)	14	85.7	6.5 (4.3-9.9)	43.4 (19.3-97.4)	15	86.7	6.8 (4.2-10.9)	35.0 (16.4-74.5)	14	50.0	3.1 (2.3-4.2)	8.1 (3.5-19.0)
No	91	92.3	20.9 (16.9-25.9)	215.5 (155.7-298.3)	105	98.1	14.8 (12.7-17.2)	94.9 (79.5-113.4)	145	85.5	8.0 (7.0-9.1)	52.6 (41.5-66.8)	175	80.0	5.9 (5.3-6.7)	27.1 (21.8-33.5)	186	63.4	4.2 (3.8-4.6)	13.9 (11.1-17.4)
Chronic conditions																				
Yes	9	88.9	20.7 (8.6-50.0)	195.4 (46.4-823.5)	12	91.7	11.8 (6.9-20.3)	67.3 (29.8-151.6)	17	70.6	5.7 (3.8-8.5)	26.9 (10.8-66.6)	16	56.2	3.6 (2.5-5.2)	10.6 (4.6-24.3)	12	58.3	4.2 (2.5-7.3)	12.8 (4.3-38.4)
No	91	90.1	20.1 (16.1-25.1)	195.7 (137.4-278.8)	98	99.0	15.1 (12.9-17.6)	98.6 (83.3-116.8)	142	87.3	8.1 (7.2-9.2)	55.9 (44.4-70.4)	174	82.8	6.3 (5.6-7.1)	30.2 (24.5-37.1)	188	62.8	4.1 (3.7-4.5)	13.4 (10.8-16.7)
Interval between 2 doses (days)																				
21-27	87	88.5	18.2 (14.4-22.9)	169.9 (116.4-248.0)	94	97.9	14.7 (12.5-17.3)	94.0 (77.6-113.8)	6	66.7	6.9 (2.3-21.1)	30.2 (3.2-288.2)	8	75.0	4.4 (2.7-7.1)	18.1 (5.6-58.2)	14	64.3	3.7 (2.7-5.0)	12.5 (5.5-28.5)
28-35	13	100.0	40.3 (28.7-56.7)	504.1 (358.7-708.5)	16	100.0	14.7 (10.2-21.2)	98.2 (68.2-141.5)	153	86.3	7.9 (7.0-8.9)	52.8 (42.1-66.2)	182	80.8	6.1 (5.4-6.8)	28.1 (22.8-34.7)	186	62.4	4.1 (3.7-4.6)	13.5 (10.8-16.9)

*(+)% means NT_50_ positivity rate for each group.

Among participants vaccinated with BBIBP-CorV, 100 had blood samples collected at 1 month after the second dose (median 34 days, IQR 32-34 days); 111 at 3 months (92 days, IQR 90.5-94); 162 at 6 months (186 days, IQR 183-186); 195 at 9 months (273 days, IQR 273-274); and 200 at 12 months (366 days, IQR 362-366). Among participants vaccinated with CoronaVac, 100 had blood samples drawn at 1 month (30 days, IQR 30-32); 110 at 3 months (93 days, IQR 92-96); 159 at 6 months (184 days, IQR 183-184); 190 at 9 months (275 days, IQR 274-275); and 200 at 12 months (364 days, IQR 362-366 days).

We obtained 62 blood samples from recovered COVID-19 patients who had been infected with the SARS-CoV-2 D614G variant six months previously. These survivors ranged in age from 18 to 58 years, with a mean age of 37.5 (sd 12.1 years); 37 (59.7%) were male (**Table 2**).

**Table 2:** Participant characteristics and nAb responses to COVID-19 convalescent sera obtained 6 months after diagnosis.

	Number of Participants (%)	Positive rate (%)*	Geometric mean titers		Geometric mean concentration (IU/mL)	
			GMTs	95%*CI*	GMC	95%*CI*
Sex						
Male	37(59.7)	94.6	12.9	9.5-17.4	103.9	68.8-156.9
Female	25(40.3)	96.0	14.0	10.6-18.5	116.7	77.2-176.3
Age group (at diagnosed time, years)						
18-29	20(32.3)	95.0	13.6	9.3-19.8	110.5	64.4-189.6
30-39	15(24.2)	100.0	15.1	10.6-21.5	137.0	96.2-194.9
40-49	14(22.6)	92.9	12.8	7.5-21.8	99.6	45.7-216.9
50-59	13(21.0)	92.3	11.7	6.6-20.9	89.9	38.8-208.4
Total	62(100.0)	95.2	13.3	10.8-16.4	108.9	81.5-145.4

*Positive rate (%) means NT_50_ positivity rate for each group.

### NAb responses

The primary immunogenicity outcomes were NT_50_ positivity rates, geometric mean titers (GMTs), and geometric mean concentrations (GMC, IU/mL) of neutralizing antibodies. NT_50_ seropositivity was defined as a neutralizing antibody titer of at least 1:4. A seropositivity threshold of 1:4 has been used as a measure of seropositivity in live SARS-CoV-2 virus neutralization tests in published clinical trials of COVID-19 vaccines (16–18).

**Table 1** shows NT_50_ positivity rates and geometric mean titers (GMTs) and geometric mean concentrations (GMC, IU/mL) of neutralizing antibodies by selected characteristics of participants. NT_50_ positive rates for BBIBP-CorV declined from 98.0% at 1 month to 53.5% at 12 months (trend χ^2^ = 109.0, *P*<0.001), with corresponding nAb titers declining from 25.0 [95%CI: 21.3-29.4] to 4.0 [95%CI: 3.5-4.4], an 84.0% decrease; nAb concentrations declined from 217.2 [95%CI: 178.5-264.3] to 10.9 [95%CI: 8.7-13.8] (IU/mL). NT_50_ positivity rates for CoronaVac declined from 90.0% at 1 month to 62.5% at 12 months (trend χ^2^ = 56.3, *P*<0.001) with corresponding nAb titers declining from 20.2 [95%CI: 16.3-24.9] to 4.1 [95%CI: 3.7-4.5], a 79.7% decrease; nAb concentration declined from 195.7 [95%CI: 139.5-274.5] to 13.4 [95%CI: 10.8-16.6] (IU/mL).

**Figure 2:**
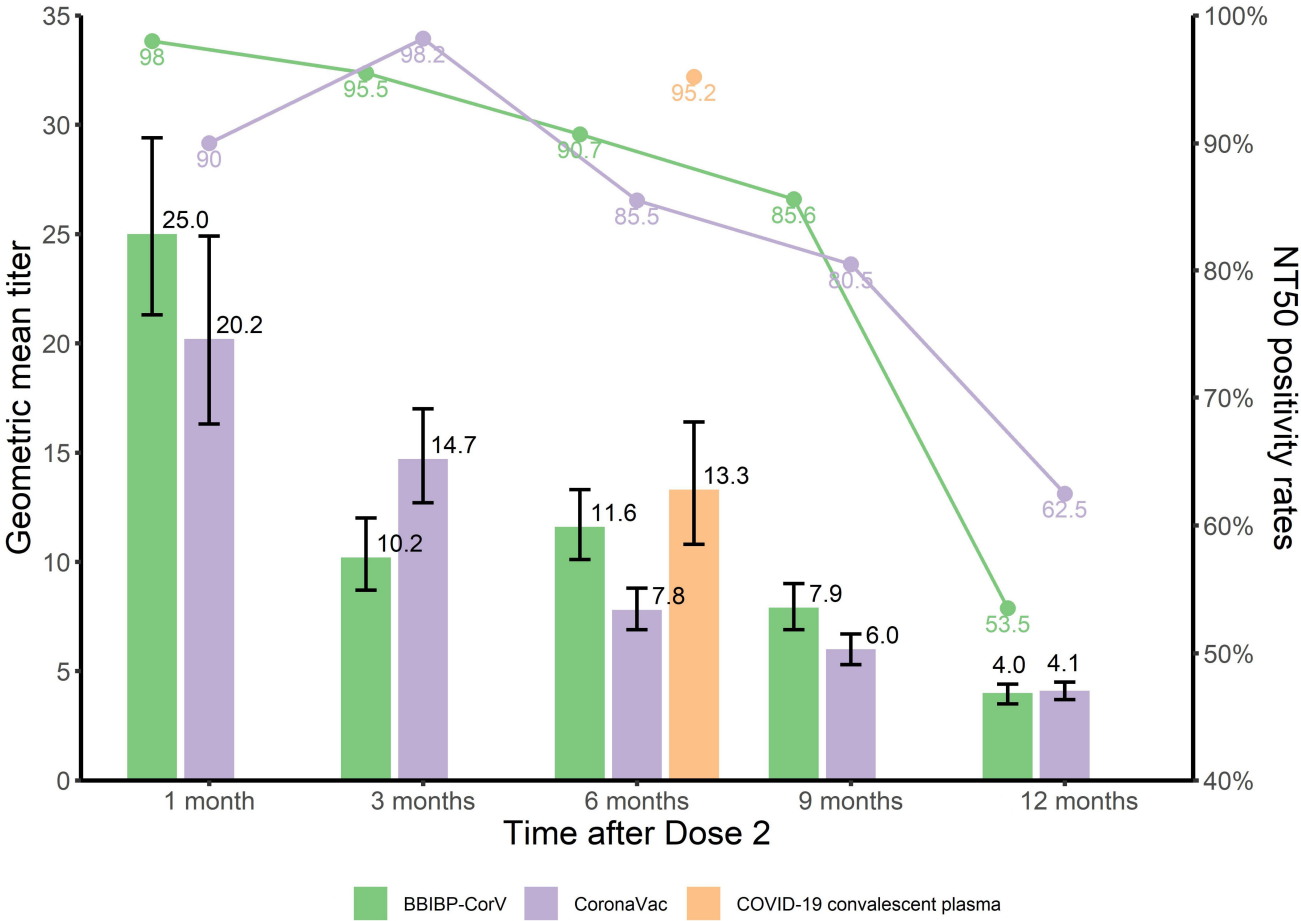
NT_50_ positivity rates and GMTs of nAb at different time points after immunization with inactivated COVID-19 vaccine and COVID-19 convalescent sera.

**Figure 3:**
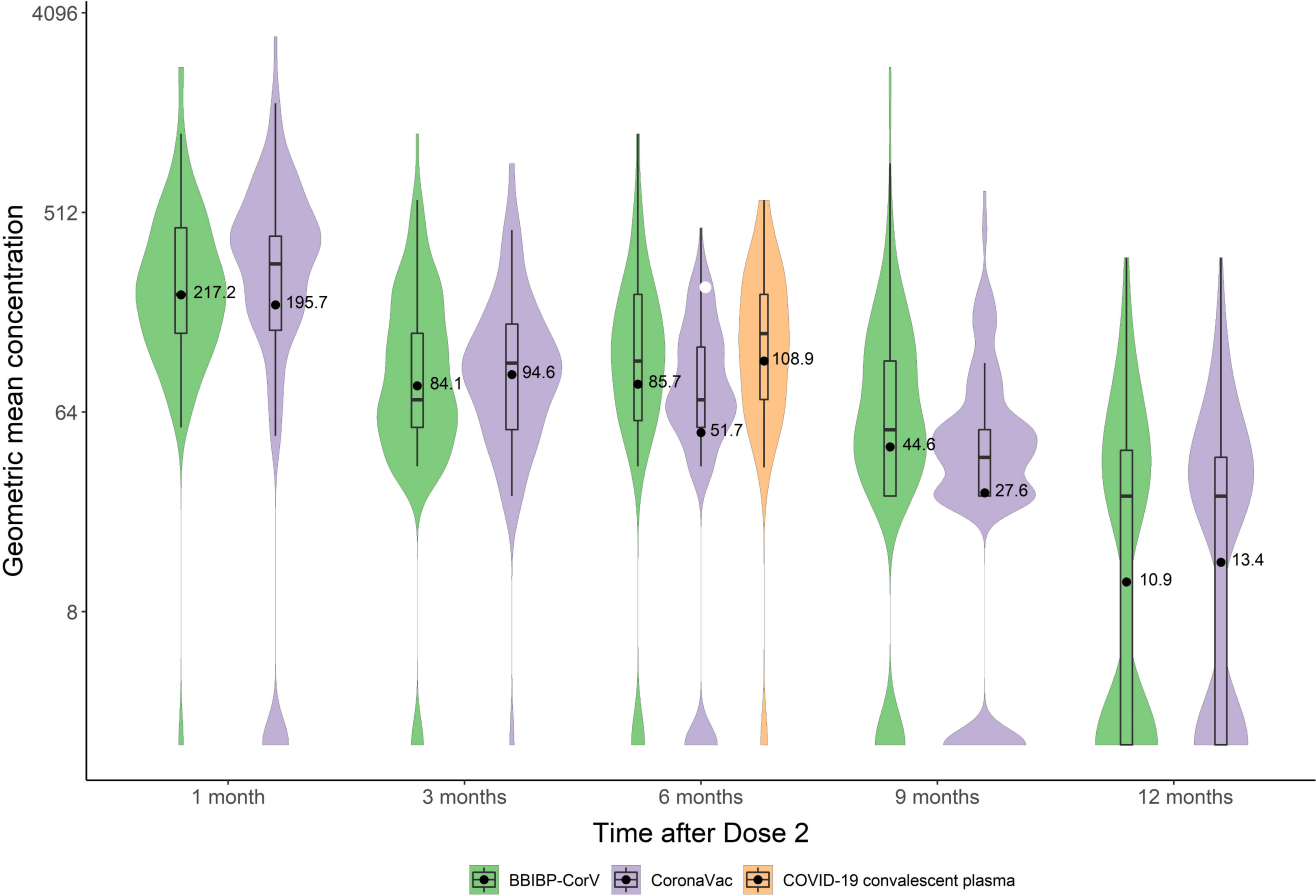
GMCs of nAb at different time points after immunization with inactivated COVID-19 vaccine and COVID-19 convalescent sera.

**Table 2** shows NT_50_ positivity rates and GMTs and GMC (IU/mL) of neutralizing antibodies in people who recovered from SARS-CoV-2 infection. Six months after infection, the NT_50_ positive rate was 95.2%, the GMT was 13.3 [95%CI: 10.8-16.4], and the GMC was 108.9 [95%CI: 81.5-145.4] IU/mL. Kruskal Wallis testing showed that GMCs of these survivors were comparable to the GMCs of BBIBP-CorV-vaccinated subjects at 6 months (*P*=0.39), and were slightly greater than GMCs for CoronaVac at 6 months (*P*<0.001) and comparable to CoronaVac at 3 months (*P*=0.07) (**Figures 2**, **3**).

### Multivariable analyses of nAb titers

Logistic regression was used to investigate the association of sociodemographic and immunization-related factors with NT_50_ positivity rates. Age and time since the second dose were associated with NT_50_ positivity for both BBIBP-CorV and CoronaVac. NT_50_ positivity rates were higher for females vaccinated with BBIBP-CorV (OR=1.669, *P*=0.020) but not for females vaccinated with CoronaVac. Comorbidities were associated with a decline of NT_50_ positivity for CoronaVac (OR=0.453, *P*=0.018) but not for BBIBP-CorV. There were no statistically significant associations between NT_50_ positivity and obesity or inter-dose interval. A logistic regression model that included these two vaccines showed no statistically significant association between NT_50_ positivity and the brand of vaccine (OR=0.909, *P*=0.512).

Multiple linear regression on log-transformed GMCs was used to investigate associations of sociodemographic and immunization-related variables with GMCs. Time since the second dose was associated with lower GMCs for both BBIBP-CorV and CoronaVac. For BBIBP-CorV, GMCs were lower among 40-49-year-olds (*P*=0.007) and 50-59-year-olds (*P*<0.001) than 18-29-year-olds. For CoronaVac, only 50-59-year-olds had lower GMCs than 18-29-year-olds (*P*=0.018). Being female was associated with a slower GMCs decline for BBIBP-CorV but not for CoronaVac. Presence of comorbidity was associated with more rapid decline of GMCs; longer inter-dose interval was associated with slower GMC decline for CoronaVac but not for BBIBP-CorV. Obesity was not statistically significantly associated with GMCs for either vaccine. A multiple linear regression model that included these two vaccines showed slighter lower GMCs with CoronaVac than BBIBP-CorV 6 months after completion of the primary series (*P*=0.010). Full regression model results are shown in **Tables 3**, **4**.

**Table 3:** Logistic regression analyses of NT_50_ positivity rates of nAb.

Variable	BBIBP-CorV (n=768)		CoronaVac (n=759)	
	OR (95%*CI*)	*P* value	OR (95%*CI*)	*P* value
(Intercept)	63.103 (17.905-403.491)	< 0.001	15.042 (6.712-37.061)	<0.001
Sex				
Male	Ref		Ref	
Female	1.669 (1.089-2.577)	0.020	1.055 (0.706-1.575)	0.793
Age group				
18-29y	Ref		Ref	
30-39y	1.045 (0.557-1.973)	0.890	0.493 (0.268-0.879)	0.019
40-49y	0.630 (0.349-1.128)	0.122	0.817 (0.438-1.490)	0.516
50-59y	0.398 (0.217-0.720)	0.003	0.487 (0.248-0.939)	0.034
Obesity(BMI≥28 kg/m^2^)				
No	Ref		Ref	
Yes	0.705 (0.376-1.355)	0.283	0.795 (0.405-1.636)	0.516
Chronic conditions				
No	Ref		Ref	
Yes	1.129 (0.567-2.331)	0.735	0.453 (0.238-0.883)	0.018
Time after 2nd dose				
1 month	Ref		Ref	
3 months	0.410 (0.057-1.971)	0.296	6.528 (1.651-43.421)	0.018
6 months	0.230 (0.033-0.957)	0.072	0.384 (0.133-1.061)	0.070
9 months	0.142 (0.021-0.561)	0.014	0.256 (0.091-0.683)	0.008
12 months	0.025 (0.004-0.093)	< 0.001	0.103 (0.038-0.265)	<0.001
Interval between 2 doses (days)				
21-27	Ref		Ref	
28-35	0.783 (0.414-1.441)	0.442	1.786 (0.814-3.868)	0.143

**Table 4:** Multiple linear regression analyses of GMCs of nAb.

Variable	BBIBP-CorV (n=768)		CoronaVac (n=759)	
	Estimate (95%*CI*)	*P* value	Estimate (95%*CI*)	*P* value
(Intercept)	2.353 (2.204 - 2.502)	< 0.001	2.372 (2.210 - 2.535)	< 0.001
Sex				
Male	Ref		Ref	
Female	0.200 (0.113 - 0.288)	< 0.001	0.032 (-0.059 - 0.122)	0.494
Age group				
18-29y	Ref		Ref	
30-39y	-0.034 (-0.154 - 0.086)	0.575	-0.122 (-0.250 - 0.007)	0.063
40-49y	-0.164 (-0.283 - -0.045)	0.007	-0.056 (-0.185 - 0.074)	0.397
50-59y	-0.226 (-0.351 - -0.101)	< 0.001	-0.174 (-0.318 - -0.030)	0.018
Obesity(BMI≥28 kg/m^2^)				
No	Ref		Ref	
Yes	-0.078 (-0.220 - 0.065)	0.284	-0.081(-0.253 - 0.090)	0.354
Chronic conditions				
No	Ref		Ref	
Yes	0.103 (-0.053 - 0.259)	0.196	-0.183 (-0.347 - -0.019)	0.029
Time after 2nd dose				
1 month	Ref		Ref	
3 months	-0.425 (-0.588 - -0.262)	< 0.001	-0.311 (-0.480 - -0.143)	< 0.001
6 months	-0.383 (-0.567 - -0.199)	< 0.001	-0.736 (-0.947 - -0.526)	< 0.001
9 months	-0.656 (-0.838 - -0.475)	< 0.001	-1.017 (-1.223 - -0.810)	< 0.001
12 months	-1.273 (-1.450 - -1.096)	< 0.001	-1.327 (-1.529 - -1.125)	< 0.001
Interval between 2 doses(days)				
21-27	Ref		Ref	
28-35	-0.047 (-0.176 - 0.081)	0.472	0.179 (0.009 - 0.349)	0.039

The total n of analyzed subjects for BBIBP-CorV was 768. F=39.78; P < 0.001; adjusted R^2^ = 0.357. The total n of analyzed subjects for CoronaVac was 759. F=29.15; *P* < 0.001; adjusted R^2^ = 0.290.

## Discussion

Our study of post-vaccination neutralizing antibody levels showed that under real-world conditions with no exogenous boosting by SARS-CoV-2 infection, BBIBP-CorV and CoronaVac inactivated COVID-19 vaccines were immunogenic, inducing seropositivity against ancestral SARS-CoV-2 in more than 90% of adults and maintaining seropositivity for 12 months for more than half of the 12-month post-vaccination subjects. Neutralizing antibody titers and geometric mean concentrations were highest among one- to three-month post-vaccination subjects and were similar to 6-month post-infection convalescent nAb levels among the three- to six-month post-vaccination subjects. Among 12-month post-vaccination subjects, nAb levels were low. Declining neutralizing antibody levels is consistent with a need for booster vaccination approximately six months after primary series vaccination.

Many vaccines have been developed for controlling the epidemic (19, 20) and several are in large-scale global use (21, 22). As of the end of 2021, more than 9 billion doses of COVID-19 vaccines have been administered globally, with inactivated vaccine CoronaVac and BBIBP-CorV comprising 41% of the doses administered (5, 23). Full-series coverage of COVID-19 vaccines in China was 80% by the end of 2021, and the vast majority (86%) of doses administered have been of these two inactivated vaccines (15). With widespread use of these vaccines, it is essential to understand changes in seropositivity and nAb levels over 12 months.

We found that BBIBP-CorV and CoronaVac had similar nAb kinetics and persistence. Zeng and colleagues found that CoronaVac immunity endures for 6 months, with nAb GMT 6 months after two doses of 6.8 [95%CI: 5.2-8.8] and seropositivity of 35% (19/54, with 1:8 as positive) (10). Jingxin Li and colleagues showed that GMT was 2.2-2.5 at 3-6 months after 2 doses of CoronaVac (24). Results from their pre-enhanced neutralization antibody response levels were slightly lower than what we found. Differences may be due to differences in test methods or conditions, as standardization of nAb testing is challenging and has not yet been achieved globally.

Previous studies have shown that nAb levels decrease over time after initial vaccination with COVID-19 vaccines, regardless of vaccine type, and showing substantial decrease by 6 to 8 months. The rate and magnitude of decline varies significantly by technical vaccine platform (7, 8, 25). For example, nAb from Moderna’s mRNA-1273 vaccine persists 3-6 months after second dose with all participants sustaining detectable activity and with ID_50_ GMTs of 775 [95% CI: 560-1071] at 3 months and 361 [95% CI: 258-504] at 6 months among 18-to-55-year-olds (8, 26, 27). Following vaccination with Pfizer BNT162b2 vaccine, neutralizing antibodies declined similarly (28). A study from Israel among health care workers showed that GMTs decreased from 557.1 to 119.4 in 6 months (7, 29). In our study, seropositivity remained above 85% and GMTs remained 8 or above (1:4 considered as positive) with either BBIBP-CorV or CoronaVac for 6 months after completion of full-series vaccination – lower GMTs than other types of COVID-19 vaccines, especially mRNA vaccines.

There are breakthrough infections from the current SARS-CoV-2 variants for all COVID-19 vaccines, regardless of platform (30–32). As has been the case with other COVID-19 vaccines, inactivated vaccines have been shown to be effective for reducing severe illness and death. Evaluation in China showed no statistically significant difference in effectiveness between BBIBP-CorV and CoronaVac (22, 33). However, more evidence is needed on levels of neutralizing antibody necessary for protection and the role of immune responses other than humoral immunity for protection.

Khoury and colleagues analyzed data from seven mainstream vaccines and convalescent cohorts, and found that the higher the ratio of neutralizing antibodies generated after vaccination compared with convalescent levels, the higher the protective rate of the vaccine. She and her colleagues estimated that a neutralization level for 50% protection against detectable SARS-CoV-2 infection is 20.2% of the mean convalescent level (95% CI: 14.4–28.4%) (34). Previous reports have shown that inactivated vaccines were less immunogenic than natural infection (35), while we found that neutralizing immunity induced by BBIBP-CorV and CoronaVac at 6 months and 9 months after vaccination were similar to or slightly lower than convalescent sera 6 months after natural infection based on seropositivity and GMCs. Our finding is consistent with findings by Wang and colleagues using wild-type pseudovirus assays (36), perhaps because most of the recovered serum used in our study came from patients with mild or asymptomatic infection.

Using regression models, we found that nAb seropositivity and GMCs of both inactivated vaccines decreased with age, especially in the 50-59 age group, similar to findings in other reports (7, 37, 38). Antibody levels following BBIBP-CorV vaccine were higher in women than in men, which is similar to results with BNT162b2 mRNA vaccine (7). Obesity had no significant effect on nAb levels in our study, however, several studies have showed that SARS-CoV-2 neutralizing antibodies are positively associated with BMI (39), and obesity increases risk for hospitalization, ICU admission, and death among patients with COVID-19 (40). In our study, only CoronaVac was found to have an association between comorbidities and immunogenicity, a finding that was also observed in a study from Chile (41). It is not clear why there are differences in the immune responses to these two vaccines by gender and comorbidity and whether these differences are sustained in other studies and with other variants. Differences may be related to the survey population, the sample size, or differences the vaccines, and will require further study to evaluate.

Although neutralizing antibodies are known to be potential correlates of protection against COVID-19 (42), comparison of immunological data is often challenged by differences in assays, target antigens, numerical readouts, and endpoints. The WHO international standard for anti-SARS-CoV-2 immunoglobulin is expected to standardize measurements for clinical trial results of neutralization assays from different laboratories (34). In our study, we used the first WHO IS, NIBSC code 20/136 to facilitate comparison of SARS-CoV-2 neutralization assay data from multiple assay formats and vaccine candidates. The WHO IS 20/136 standard was established by the WHO Expert Committee on Biological Standardization in December, 2020. Wider use of the WHO IS will facilitate vaccine evaluations.

A recent study conducted by the Oxford COVID vaccine trial group reported potential correlates of protection against symptomatic and asymptomatic SARS-CoV-2 infection, using WHO international standard units for all assays (43). This study demonstrated that the AZD1222 vaccine efficacy of 80% against symptomatic infection with B.1.1.7 variant of SARS-CoV-2 was achieved with 26 (95% CI: NC, NC) international unit IU/mL and 247 (95% CI: 101, NC) normalized neutralization titers (NF_50_) for pseudovirus and live-virus neutralization, respectively. With live virus neutralization assays, vaccine efficacy of 80% against symptomatic was achieved with 120 IU/mL (95% CI: 56 to 298), which was lower than GMCs at 1 month for both the inactivated vaccines in our study (BBIBP-CorV 217.2 IU/mL, and CoronaVac 195.7 IU/mL), but higher than that at 3 months (BBIBP-CorV 84.1 IU/mL, and CoronaVac 94.6 IU/mL). To our knowledge, our study is the first to report neutralization titer results post BBIBP-CorV and CoronaVac vaccination in IU/mL, facilitating assay-to-assay comparisons for future studies.

One of the strengths of our study is providing a measure of pure vaccine induced immunity, without exogenous booster by natural infection. This was possible because there were no large COVID-19 outbreaks in mainland China during the study period and no participants had history of infection with SARS-CoV-2. A second strength is that we had sufficient sample sizes to provide immunity estimates for 12 months separately for both BBIBP-CorV and CoronaVac, and with reasonably narrow CIs. Third, our study used WHO 1st international standard (NIBSC code 20/136) for assay immunogenicity, allowing comparison with other studies.

Our study has several limitations. First, we used a cross-sectional observation study design, rather than a longitudinal study of the same cohort. Although this limits the precision of results, a cross-sectional study, especially one conducted in an environment without exogenous boosting, represents a real-world study of pure vaccine-induced population immunity over time. Second, our study analyzed neutralizing antibodies and not individual antigens. We did not evaluate cellular immunity, which has been provided by other studies. Third, due to the limited sample size, our study could not conduct analyses of subgroups with specific comorbidities and we used a small number of covariates in our analyses. Fourth, our study did not include children or subjects over 60 years, limiting generalizability. Fifth, neutralizing antibody levels were tested against ancestral SARS-CoV-2 only, precluding results and conclusions related to variants of concern such as Omicron.

Our study has implication for immunization strategy. Over 85% of people in China have completed their primary vaccination series, and the vast majority (86%) of doses administered have been these two inactivated vaccines. Real-world evidence of waning markers of immunity supports booster dose programs to enhance and maintain immunity. We should continue monitoring population immunity and assess protection effectiveness of China’s vaccines during outbreaks to provide information that will be useful for updating immunization strategy.

## Conclusion

In conclusion, we found similar kinetics of post-vaccination nAb levels of BBIBP-CorV and CoronaVac along with prominent immune persistence (seropositivity persisted for at least 12 months). Although nAb levels decreased rapidly, neutralizing immunity induced by BBIBP-CorV six months and CoronaVac three months postvaccination were comparable with six-month convalescent sera from individuals recovering from COVID-19. Future research will focus on immunity of inactivated vaccine booster doses and optimization of population immunity in China.

## Data availability statement

The raw data supporting the conclusions of this article will be made available by the authors, without undue reservation.

## Ethics statement

This study was reviewed and approved by Medical Ethics Committee of The Chinese Center for Disease Control and Prevention (Approval notice: 202101). The patients/participants provided their written informed consent to participate in this study.

## Author contributions

The study was conceived and designed by WT and ZY. Data analysis were performed by FW, XW, LT, QL and BH, YD, LZ, FY, WW made laboratory testing. HL, LF, WR, HJZ, SC, YL, FC were responsible for data collection, FW, XW, LT, QL, HZ and DW wrote the first draft, ZA, FG and LR contributed to the final version of the paper. All authors contributed to the article and approved the submitted version.

## Funding

This work was supported by the Chinese Center for Disease Control and Prevention Research Founding (JY21-3-01), the National Key Research and Development Program of China (2021YFC2301600), and the National Natural Science Foundation of China (Grant 82041021 and Grant 82061138008).

## Acknowledgments

We gratefully thank the fellow from Zhejiang CDC, Shanxi CDC, for participants recruitment and blood sample collection.

## Conflict of interest

The authors declare that the research was conducted in the absence of any commercial or financial relationships that could be construed as a potential conflict of interest.

## Publisher’s note

All claims expressed in this article are solely those of the authors and do not necessarily represent those of their affiliated organizations, or those of the publisher, the editors and the reviewers. Any product that may be evaluated in this article, or claim that may be made by its manufacturer, is not guaranteed or endorsed by the publisher.
